# Shared and Distinctive Ultrastructural Abnormalities Expressed by Megakaryocytes in Bone Marrow and Spleen From Patients With Myelofibrosis

**DOI:** 10.3389/fonc.2020.584541

**Published:** 2020-11-16

**Authors:** Maria Zingariello, Vittorio Rosti, Alessandro M. Vannucchi, Paola Guglielmelli, Maria Mazzarini, Giovanni Barosi, Maria Luisa Genova, Anna Rita Migliaccio

**Affiliations:** ^1^Unit of Microscopic and Ultrastructural Anatomy, Department of Medicine, University Campus Bio-Medico, Rome, Italy; ^2^Center for the Study of Myelofibrosis, Laboratory of Biochemistry Biotechnology and Advanced Diagnosis, IRCCS Policlinico San Matteo, Pavia, Italy; ^3^CRIMM; Center Research and Innovation of Myeloproliferative Neoplasms, AOUC, University of Florence, Florence, Italy; ^4^Biomedical and Neuromotor Sciences, Alma Mater University Bologna, Bologna, Italy; ^5^Myeloproliferative Neoplasm-Research Consortium, New York, NY, United States

**Keywords:** myeloproliferative neoplasms, myelofibrosis, megakaryocytes, ultrastructural analyses, cell metabolism

## Abstract

Numerous studies have documented ultrastructural abnormalities in malignant megakaryocytes from bone marrow (BM) of myelofibrosis patients but the morphology of these cells in spleen, an important extramedullary site in this disease, was not investigated as yet. By transmission-electron microscopy, we compared the ultrastructural features of megakaryocytes from BM and spleen of myelofibrosis patients and healthy controls. The number of megakaryocytes was markedly increased in both BM and spleen. However, while most of BM megakaryocytes are immature, those from spleen appear mature with well-developed demarcation membrane systems (DMS) and platelet territories and are surrounded by platelets. In BM megakaryocytes, paucity of DMS is associated with plasma (thick with protrusions) and nuclear (dilated with large pores) membrane abnormalities and presence of numerous glycosomes, suggesting a skewed metabolism toward insoluble polyglucosan accumulation. By contrast, the membranes of the megakaryocytes from the spleen were normal but these cells show mitochondria with reduced crests, suggesting deficient aerobic energy-metabolism. These distinctive morphological features suggest that malignant megakaryocytes from BM and spleen express distinctive metabolic impairments that may play different roles in the pathogenesis of myelofibrosis.

## Introduction

Megakaryocytes are specialized cells of the bone marrow (BM) derived from committed hematopoietic progenitor cells that become endorsed with the ability to produce platelets through a complex process of terminal maturation defined thrombopoiesis. During maturation, megakaryocytes retain the ability to replicate their genomic information while becoming unable to undergo cytodieresis and undergoing instead several cycles of endomitosis. Therefore, as they mature, megakaryocytes progressively increase in size and acquire nuclei larger than normal and with poly-lobulated morphology (1, 2). In addition, during the process of terminal maturation, the cytoplasm of the megakaryocytes acquires distinctive ultrastructural features described for the first time by Dr. Zucker-Franklin (1, 3). These changes involve massive compartmentalization of the cytoplasm into discrete regions delimited by intrusions of the plasma membrane which forms a reticulum defined as demarcation membrane system (DMS) delimiting areas (the pro-platelet territories) embedded with granules containing platelet-specific proteins, such as pro-hemostatic factors (fibrinogen, fibronectin, von Willebrand factor), adhesion receptors (von Willebrand factor and P-selectin), pro-angiogenic (vascular endothelial growth factor and basic fibroblast growth factor), and anti-angiogenic (endostatin and thrombospondin-1) growth factors (4). On the basis of their distinctive ultrastructural morphology, megakaryocytes are classified into four stages of maturation: the pro-megakaryoblast (Stage 0), the megakaryoblast (Stage I), the promegakaryocyte (Stage II), and the mature megakaryocyte (Stage III) (**Figure S1**) (1). Recent evidence indicates that mature megakaryocytes migrate toward the sinusoids where they are stimulated by shear stresses from the blood flow and by stimuli provided by endothelial cells to re-organize their cytoskeletal network releasing platelets into the blood stream (5–7).

The adhesion proteins expressed by the megakaryocytes allow their interaction with other cells. As an example, P-selectin mediates the interaction with neutrophils that are then engulfed within the DMS by emperipolesis. Under steady-state conditions, neutrophils use emperipolesis to pass through the DMS without altering the functions of the megakaryocytes (8, 9). Inflammation, however, activates a process of pathological emperipolesis during which neutrophils fuse their plasma membrane with that of the DMS, releasing proteases in the cytoplasm of the megakaryocytes. This process leads to death of the megakaryocytes by a Tunnel-negative process defined para-apoptosis (see **Figure S1**) (10, 11). By contrast with apoptosis, in para-apoptosis chromatin does not undergo degradation but becomes tightly condensed (12) conferring to the nuclei distinctive heterochromatic features (13, 14).

Several morphological evidences, corroborated more recently by molecular data, indicate that the malignant megakaryocytes drive the progression of Philadelphia-negative myeloproliferative neoplasms to their end stage, myelofibrosis (MF) (15). More specifically: *i)* studies in mouse model with megakaryocyte restricted expression of the driver *JAK2V617F* mutation (See **Table S1** for gene and protein abbreviations) demonstrate that these cells are necessary and sufficient to induce MF (16, 17) and *ii)* both in patients and mouse models, the driver mutations induce a RSP14 ribosomopathy that reduces the GATA1 content in megakaryocytes which is responsible for their retarded maturation (18, 19).

The presence in BM of numerous clusters of megakaryocytes with ultrastructural abnormalities indicating retarded maturation was the first hallmark reported for MF (20, 21). Additional studies indicated that the ultrastructure abnormalities are consistent with a block between Stage II and III (20, 22) and with engulfment by neutrophils engaged in pathological emperipolesis which induce their death by para-apoptosis (11, 22–25). This pathological megakaryocyte-neutrophil interaction has been hypothesized to be responsible for fibrosis and hematopoietic failure by increasing the bioavailability of TGF-β in the BM microenvironment (26). Due to BM failure and increased progenitor cell trafficking, MF patients develop hematopoiesis in the spleen which also contains great numbers of megakaryocytes (27). Whether, in MF, spleen megakaryocytes express ultrastructural abnormalities similar to those of the BM cells has not been investigated as yet. The aim of our study is to compare the morphological abnormalities of megakaryocytes from BM and spleen of patients with MF.

## Material and Methods

### Human Subjects

Bone marrow and spleen samples were obtained from 5 and 11 MF patients, respectively for a total of 16 patients (8 females and 8 males; median age = 52.81 years, min 22 and max 70). BM and spleens were from different patients. Post-trauma spleen from three healthy donors (i.e. three different de-identified subjects for each tissue type, age and sex unknown) were analyzed as control (healthy controls, HC). The clinical features of the MF patients included in our study are summarized in **Table 1**. This study was approved by the institutional review board of Policlinico San Matteo, Pavia, Italy (Authorization No. 20110004143, Sept 26, 2011) and is compliant with the Declaration of Helsinki for Studies Involving Human Subjects.

**Table 1:** Clinical data of the MF patients included in this study.

ID	Diagnosis	Disease duration at harvest	Hb/WBC/Plt disease onset Hb/WBC/Plt harvest^*^	Spleen (cm from left costal margin)	Fibrosis grade	DIPSS	IPSS	Mutation (allele burden)	Therapy
**BM**									
MF1 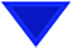	PMF	4 years	14.9/33.05/328 13.4/5.0/211	0 cm	MF2	Int-1	Int-2	JAK2 homo	Rux 20 mg ×2
MF2 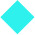	PMF	14 years	13.6/7.8/618 11.0/6.9/230	2 cm	MF2	Int-1	Low	MPL	HU 1 g/day
MF3 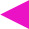	pref-MF	5 years	16.7/9.74/599 15.6/8.59/597	NP	MF0	Int-1	Int-1	JAK2 hetero	Phlebotomy and aspirin
MF4 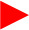	pref-MF	1 year	13.4/3.5/173 14.7/4.17/137	8 cm	MF0/1	low	low	JAK2 hetero	Dicumarolic (previous splanchnic thrombosis)
MF5 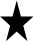	PMF	2 years	12.7/18.1.3/322 12.5/25.1/129	12 cm	MF3	Int-2	Int-1	JAK2V617 (76%)	HU 1.5 g/day
**SPLEEN**									
MF6 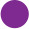	PMF	4 years	11/8.6/490 5.7/11.1/22	25 cm	MF2	Int-2	Low	JAK 2 homo	HU 1g/day
MF7 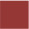	PPV-MF	6 years	NA 6.9/3.9/111	19 cm	MF2	Int-1	Int-1	JAK2 homo	HU 1g/day
MF8 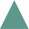	PPV-MF	2years	13.8/23.3/163.000 10.3/80.0/926.000	20 cm	MF2	Int-1	Low	JAK2V617 F (94%)	HU 1.5 g/day
MF9 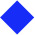	PMF	5 years	10.5/21.9/245 12.9/5.2/140	22 cm	MF2	Int-2	Int-2	JAKneg MPL/CARL unknown	HU 1g/day
MF10 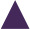	pref-MF	17 years	13.8/8.5/750 8.2/6.7/155	22 cm	MF0	Int-1	Low	JAKneg MPL/CARL unknown	HU 1/5g/day
MF11 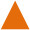	PMF	6 years	11.8/11.3/574 8.0/1.75/405	14 cm	MF1	Int-1	Low	JAK2 hetero	HU 1g/day
MF12 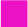	PPV-MF	3 years	15.2/6.9/266.000 12.5/4.8/605.000	15 cm	MF2	Int-1	Low	JAK2V617 F (23%)	HU 1.5 g/day
MF13 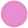	PMF	1 year	12.0/14.1/143 15.1/10.5/85	19 cm	MF3	Int-2	Int-2	JAK2 homo	HU 2g/day
MF14 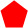	PMF	10 years	NA 2.0/8.9/104.000	25 cm	MF2	High	NA	CALR type1 (62%)	HU 1 g/day
MF15 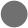	PPV-MF	2 years	14.4/15.4/354.000 8.5/19.8/196.000	30 cm	MF3	Int-2	Int-2	JAK2V617 F (100%)	HU 1 g/day
MF16 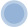	PMF	3 months	11.8/6.2/349.000 NA	20 cm	MF2	Int-2	Int-1	JAK2V617 F (50%)	None

**Figure 1:**
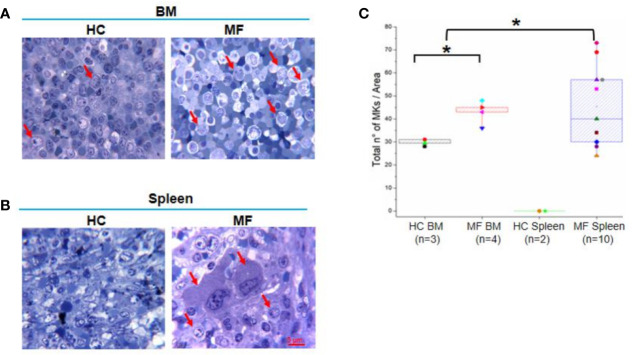
The bone marrow (BM) and spleen from myelofibrosis (MF) patients contain great numbers of megakaryocytes (MKs). Representative methylene-blue stained semi-thin sections of BM **(A)** and spleen **(B)** from MF patients and health controls (HC) (each panel a different subject). Representative megakaryocytes are indicated by red arrows. Megakaryocytes are not detected in HC spleen (over 20 semi-thin sections from two HC analyzed). Magnification 100×. Scale bar = 5μm. **(C)** Median (lines across boxes), 25–75% inter-quartile range (boxes), and maximum and minimum values (the end of the vertical line across the boxes) of the number of megakaryocytes detected in in area of 0.19 mm^2^ per semi-thin sections of multiple subjects. Values obtained with individual subjects are indicated by symbols (each subject a unique symbol, the correspondence between symbols and individual patients and HC is specified in **Table 1** and *Material and Methods*, respectively). *p < 0.05 with respect to BM from HC, n = number of subjects included in each experimental group.

Patients were diagnosed according to the 2016 World Health Organization (WHO) classification^49^. Individual patients are identified by an alphanumerical code and by a unique symbol that was used to REPORT results obtained for individual patients in the figures (see legend of **Figure 1** for further details).

PMF, primary myelofibrosis; pref-MF, prefibrotic myelofibrosis; PPV-MF, Post-Polycythemia Vera Myelofibrosis; NP, Not Palpable; Homo, homozygous; Hetero, heterozygous; Neg, negative; HU, Hydroxyurea; Rux, Ruxolitinib.

*Red fonts: “Hb/WBC/Plt at disease onset”; green fonts: “Hb/WBC/Plt at harvest”.

### Transmission-Electron Microscopy

De-calcified BM and spleen samples were fixed with 2.5% glutaraldehyde in 0.1 cacodylate buffer, pH 7.6 (Sigma) for 2 h at 4°C, post-fixed with osmium tetroxide (Sigma) for 60 min at 4°C, alcohol dehydrated and embedded in Spur resin (Poliscience, Warrington, PA, USA). Semi- and ultra-thin sections were cut with a Reichert ultramicrotome (Depew, NY, USA). Semi-thin sections were stained with Methylene-blue. Ultra-thin sections were assembled on 200 mesh copper grids and counterstained with uranyl acetate and lead citrate. Morphological observations were performed with EM 109 Zeiss (Oberkochen, Germany) and images were acquired with AXIOSKOPE microscope (ZEISS, Jena, Germany) equipped with a Coolsnap Videocamera. All quantifications were done by eye examination.

### Ultrastructural Identification Criteria

Megakaryocytes were identified on the basis of accepted criteria as depicted in **Figure S1** (1, 22): Megakaryoblasts (Stage I) are cells of 15–50 μm in diameter with large, oval or kidney-shaped nucleus and several nucleoli. The cytoplasm presents numerous ribosomes and a well-developed rough endoplasmic reticulum (RER); Pro-megakaryocytes (Stage II) are cells of 20–80 μm in diameter with irregularly shaped nucleus and relatively abundant cytoplasm containing a rudimental DMS; Mature megakaryocytes (Stage III) are >80 μm in diameter with multi-lobed nuclei surrounded by abundant cytoplasm divided into a perinuclear (with centrioles, few biosynthetically active organelles, and many granules), an intermediate (with well-developed DMS and pro-platelet territories) and a peripheral (devoid of organelles and enriched of cytoskeletal proteins and microtubules) zone (1). Pro-megakaryoblasts (Stage 0) may not be identified on the basis of ultrastructural markers. Para-apoptotic megakaryocytes are cells presenting megakaryocytic features associated with a stressed cytoplasm, DMS barely recognizable, mitochondria with broken internal crests and highly heterochromatic nuclei (**Figure S1**). Plasma and nuclear membrane abnormalities were identified on the basis of characteristic disruptions of the electron density signal of the lipid bilayer. Granules were identified as circular bodies surrounded by a single membrane enclosing electron-dense material. Polyglucosan granules and glycosomes were identified based on published TEM criteria (**Figure S2**) (28–30). Glycosomes are formed by granuli of progressively increased electron density orderly enclosing circular areas of the cytoplasm that is not surrounded by membranes. Low electron-dense acid-labile granules organize themselves as rims delimiting discrete cytoplasmic areas, the immature glycosome. These granules are turned by phosphorylation into heavy electron-dense acid-insoluble polyglucosan granules which, on the basis of their electron density, are defined as intermediate or mature glycosomes. In the case of glycogen storage diseases, acid-insoluble polyglucosan molecules accumulate in the cytoplasm either as large isolated granules or as packed glycosomes (the bodies found in Lafora disease) (30). Mitochondria are recognized as round (cross section) or rod (longitudinal section) structures surrounded by a double membrane system with the inner membrane presenting regular invagination defined crests (31).

### Data Quantification and Statistical Analyses

All evaluations were performed blind by an expert electron microscope scientist. Megakaryocyte frequency, maturation stage, para-apoptosis, and proximity to platelets/endothelial walls were evaluated in areas of semi-thin sections corresponding to 0.19 mm^2^ (one semi-thin section per subject). Additional abnormalities and their frequency were identified on individual megakaryocytes present in thin sections, the number of megakaryocytes analyzed per experimental group is specified in the legend of the Figures. Results obtained with individual donors are reported using symbol/color codes: specified for the patients in **Table 1**. Symbols used for individual healthy donors are 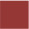, 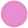, 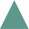 (bone marrow) and 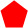, 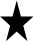
(spleen). Results are expressed as median, 25–75% inter-quartile range and maximum and minimum value P-values were calculated by ANOVA using the Origin 6.1 software (Microcal Software Inc., Northampton, MA, USA) and differences considered statistically significant with p < 0.05.

### Pathway Analyses

Genes involved in glycogen metabolism (32) and mitochondrial function were identified by computer searching using the websites https://www.genecards.org/ and https://www.kegg.jp/. The levels of their expression in microarrays of bone marrow (BM) from myelofibrosis patients *vs* healthy controls (HC) are from (33) and the original data are deposited in the Gene Expression Omnibus database, http://www.ncbi.nlm.nih.gov/geo/query/acc.cgi?acc=GSE124281.

## Results

### BM From MF Patients Contains Great Numbers of Mostly Immature Megakaryocytes While the Numerous Megakaryocytes in Their Spleen Are Mature and Surrounded by Platelets

Semi-thin sections of both BM and spleen from MF patients contain numbers of megakaryocytes 1.5-fold greater than those detected in BM from HC while megakaryocytes are barely detectable in normal spleen (**Figures 1A–C**). As previously described (20, 22), megakaryocytes in BM are small and organized in clusters (**Figure 1A**). Megakaryocytes are organized in clusters also in the spleen from these patients (**Figure 1B**). However, they are visibly two to three times larger than those observed in BM, suggesting that they are more mature.

**Figure 2:**
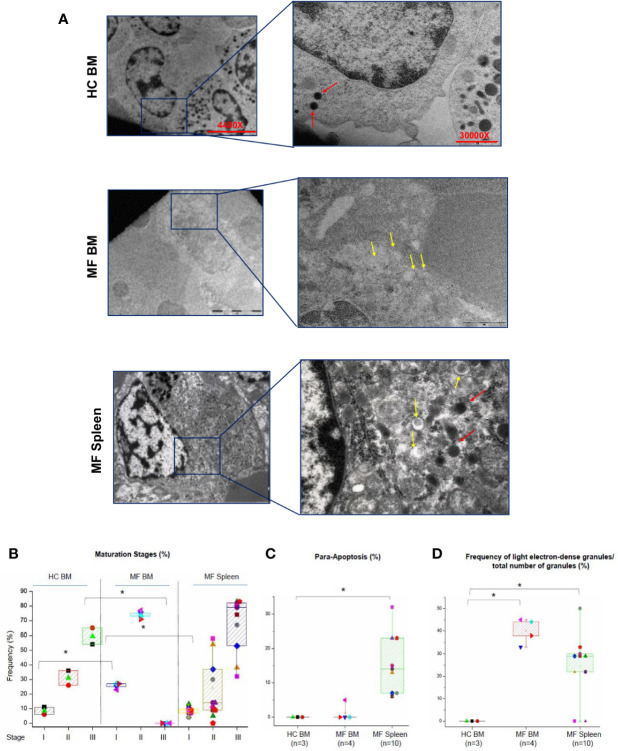
The bone marrow (BM) from MF patients contains mostly immature megakaryocytes while megakaryocytes in MF spleen are mostly mature. **(A)** Representative ultra-thin sections of Stage II megakaryocytes from BM of one healthy control (HC) and one MF patient and from a Stage III megakaryocyte from the spleen of one additional patient. Representative megakaryocytes are presented on the left (magnification 4,400×) and detail of their cytoplasm shown on the right to highlight the electron-density of their granules (magnification 30,000×). Light and heavy electron-dense granules are indicated by yellow and red arrows, respectively. Scale bars = 5 and 1 μm, as indicated. **(B)** Frequency of megakaryocytes at different stages of maturation (as percent of viable megakaryocytes) and **(C)** in para-apoptosis (as percent of total megakaryocytes) in BM and spleen from multiple MF patients and HC. *p < 0.05 with respect to the corresponding value in HC. **(D)** Frequency of light electron-dense granules with respect the total number of granules detected in megakaryocytes from HC and MF patients. *p < 0.05 with respect to the indicated experimental group. In B–C, results are presented as Median (lines across boxes), 25–75% inter-quartile range (boxes), and maximum and minimum values (the end of the vertical line across the boxes) (for further details see legend of **Figure 1**). The number of subjects included in each experimental group is indicated by n. The total number of megakaryocytes analyzed is 59 for HC BM and 215 and 430, respectively, for BM and spleen from MF patients. Results with spleen from HC are not reported because megakaryocytes are seldom detected in this organ.

The maturation profile of megakaryocytes from BM and spleen of HC and MF patients is compared in **Figures 2A, B**. In BM from HC, >55% of megakaryocytes are at Stage III with fewer cells at Stage II (~30%) or I (~10%). As expected (20, 22), in BM from MF patients, the majority of megakaryocytes are at Stage II (~75%) and I (~25%) with very few cells (<5%) at Stage III. By contrast, the maturation profile of megakaryocytes from the spleen of the patients is similar to the profile observed in BM of HC with ~65% of the cells at Stage III and few cells at Stage II (~25%) or I (~10%). Some (~15%) of the megakaryocytes detected in spleen have a clear morphology of para-apoptotic cells while para-apoptotic megakaryocytes are barely detected in BM (**Figures 2C**) which instead presented evidence of naked para-apoptotic nuclei [(24) and results not shown].

The retarded maturation expressed by megakaryocytes from BM of MF patients includes the presence in the cytoplasm of granules with light electron density, a marker for low protein content (11, 22). To further characterize the maturation profile of megakaryocytes in MF, the electron-density of granules of these cells from MF BM (Stage II) and MF spleen (Stage III) was compared to that of cells from HC BM (Stage II) (**Figures 2A, D**). While the great majority of megakaryocytes from HC BM contain high electron-dense granules, the granules of over 40% of the cells from MF BM are light. By contrast, only ~24% of megakaryocytes from MF spleen contain light electron-dense granules.

**Figure 3:**
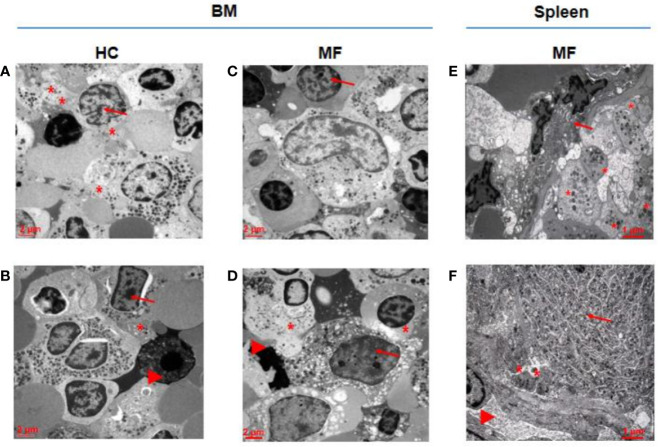
In MF spleen, megakaryocytes are frequently co-localized with platelets in the proximity of blood vessels while these cells are seldom associated with platelets in MF BM. Representative megakaryocytes from bone marrow (BM) of two HC **(A, B)** and two MF **(C, D)**, and two MF spleen **(E, F)**. Fifty percent of megakaryocytes from BM of HC and from spleen of MF patients are closely associated with platelets while the megakaryocyte shown in D is the only one out of 60 cells analyzed found to be associated with platelets in BM from these patients. Platelets, megakaryocytes, and blood vessels are indicated by *, arrows and arrowheads, respectively (for further detail see legend of **Figure 1**). Magnification 3,000× for A, B, C, D and 4,400× for E, F. Scale bars = 2 and 1 μm, as indicated.

As a surrogate measure for the potential to undergo terminal maturation, the relative physical localization between megakaryocytes and platelets within the BM and spleen architecture of MF patients and HC is compared (**Figure 3**). In HC BM, numerous Stage III megakaryocytes are found at a distance inferior of their diameter from platelets in proximity of blood vessels. By contrast, only one Stage II megakaryocyte, out of 60 cells analyzed, is found in proximity of platelets in MF BM. In the case of MF spleen, however, ~50% of Stage III megakaryocytes are found closely localized near platelets in proximity of blood vessels.

These results indicate that in MF the maturation of megakaryocytes is halted in BM but not in spleen.

### Ultrastructural Abnormalities Unique to Megakaryocytes From BM of MF Patients

The data described above indicate that megakaryocytes from BM and spleen of MF patients present common (clustered localization and poor granule content) but also distinctive (retarded maturation in BM only) ultrastructural abnormalities.

**Figure 4:**
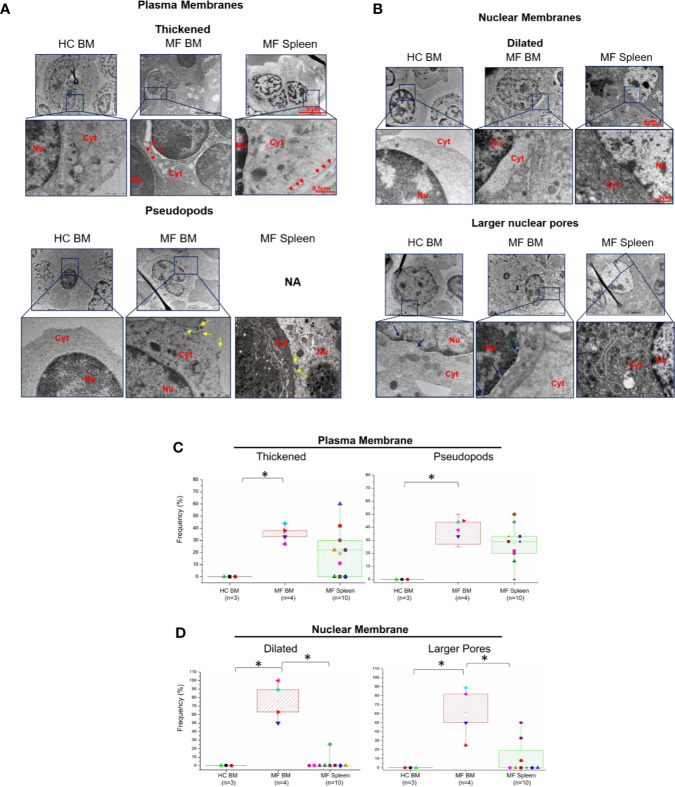
The membranes of megakaryocytes from BM of MF patients show unique ultrastructural abnormalities suggesting impaired membrane biosynthesis. Details of the plasma **(A)** and nuclear **(B)** membranes of representative megakaryocytes from BM and spleen of one control (HC) and one MF patient. The plasma membrane of megakaryocytes from MF BM appears thick and with numerous pseudopods while the nuclear membrane is dilated and with large nuclear pores. Red arrowheads indicate thick plasma membranes, yellow and blue arrows indicate pseudopods and large nuclear pores, respectively. Nu, nucleus, Cyt, cytoplasm. N.A., not available. Magnification 5,800× for megakaryocytes, 37,000× and 23,000× for the details of the plasma and nuclear membranes, respectively. Scale bars = 5 μm (5,800×), 1 μm (23,000×), and 0.5 μm (37,000×). **(C, D)** Frequency of megakaryocytes presenting ultrastructural abnormalities of plasma and nuclear membranes with respect to the total number of viable megakaryocytes analyzed per experimental group. Results are presented as median (lines across boxes), 25–75% inter-quartile range (boxes), and maximum and minimum values (the end of the vertical line across the boxes); see legend of **Figure 1** for further detail. The number of subjects included in each experimental group is indicated by n and the average number of megakaryocytes analyzed per group was 6 for HC BM, 35 for MF BM, and 91 for MF spleen. *p < 0.05 with respect to the indicated value.

**Figure 5:**
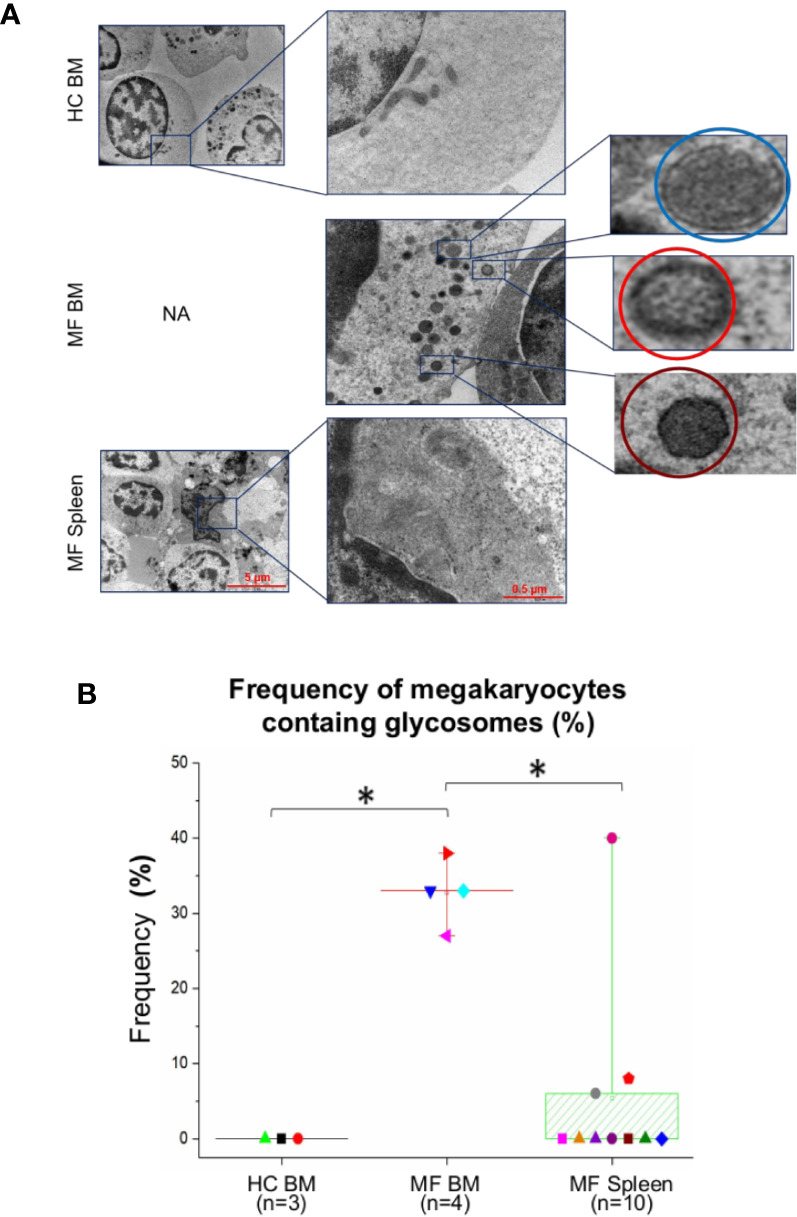
The cytoplasm of numerous megakaryocytes from bone marrow (BM) of MF patients contains structures with ultrastructural features resembling glycosomes at various stages of maturation. **(A)** Representative megakaryocytes (magnification: 5,800×, scale bar = 5 μm) from the various experimental groups with corresponding enlarged details (magnification: 37,000×, scale bar = 0.5 μm) of their cytoplasm, as indicated. The circles in the enlarged panel of MF BM indicate glycosomes composed by granules with the electron density characteristics of immature-acid soluble (blue) and mature acid-insoluble (red) polyglucosan granules and of a glycosome resembling a Lafora body (see **Figure S2** for further detail). **(B)** Frequency, as median (lines across boxes), 25–75% inter-quartile range (boxes), and maximum and minimum values (the end of the vertical line across the boxes) of megakaryocytes presenting glycogen droplets in the three groups. n = number of subjects included in each experimental group (see legend of **Figure 1** for further detail). The number of cells analyzed per group is reported in the legend for **Figure 4**. HC, healthy control; NA, not available.

Additional abnormalities found to be unique to megakaryocytes from BM of MF patients are represented by alterations of the morphological features of the plasma and nuclear membranes (**Figure 4**) and by the presence of numerous glycosomes in the cytoplasm (**Figure 5**). In fact, great numbers (~35%) of megakaryocytes from MF BM present a thick plasma membrane with numerous pseudopods that likely represent endosomes in the process of being released from the cytoplasm (**Figures 4A, C**). In addition, megakaryocytes with dilated nuclear membranes (~80% of the cells) and abnormally large nuclear pores (~65% of the cells) are also observed in BM from these patients (**Figures 4B, D**). On the contrary, these abnormalities are seldom detected in megakaryocytes from BM of HC and, with the exception of pseudopods which are indeed expressed by ~30% of the cells, they are expressed only by few (~5-20%) megakaryocytes from MF spleen.

Interestingly enough, the cytoplasm of >30% of megakaryocytes from BM of the patients contains oval structures surrounded by a rim of electron-dense material enclosing areas with homogeneous electron-density, which distinguish them from the translucent core that instead characterizes lipid droplets (**Figure 5**). The electron density and the morphological features of these structures are similar to that of the glycogen droplets present in muscle and liver cells (34). In some cases, these granules are so heavily electron-dense to resemble glycogen granules containing acid-insoluble polyglucosan formed in Lafora disease (30) (**Figures 5** and **S2**). By contrast, these structures are observed in only 5–7% of megakaryocytes from the patients’ spleen and are seldom detected in cells from BM of HC (**Figure 5**).

### Ultrastructural Abnormalities Unique to Megakaryocytes From the Spleen of MF Patients Include Altered Mitochondria

**Figure 6:**
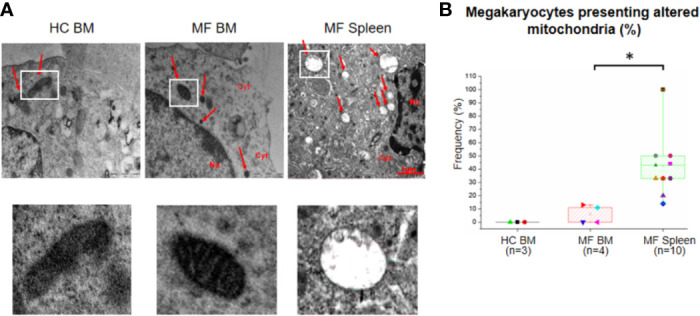
The unique ultrastructural abnormalities expressed by megakaryocytes from spleen of myelofibrotic (MF) patients include presence of altered mitochondria with disorganized inner membrane and reduced crests. **(A)** Representative megakaryocyte mitochondria are indicated by white squares. Magnification 18,500×. Scale Bars = 1 μm as indicated. **(B)** Frequency as median (lines across boxes), 25–75% inter-quartile range (boxes), and maximum and minimum values (the end of the vertical line across the boxes) of megakaryocytes with altered mitochondria (see legend of **Figure 1** for further detail). The number of megakaryocytes analyzed is 7 for BM from HC, 28 for BM, and 91 for spleen of MF patients. Statistically different (p < 0.01) values are indicated by *.

The number and morphology of mitochondria in megakaryocytes from MF BM are overall normal compared to BM from HC (**Figure 6**). By contrast, a great number (~40%) of megakaryocytes from MF spleen is characterized by the presence of altered mitochondria with disorganized internal membranes and reduced crests.

## Discussion

Our results indicate that megakaryocytes from BM and spleen from MF patients express common but also distinctive abnormalities. The number of patients analyzed (5 for BM and 11 for spleen) is relatively robust for TEM studies and make us confident that these abnormalities are present in all the patients independently from their driver mutation. Therefore, it is conceivable that they are driven by the different supportive microenvironment cues of the two organs. These results are in line with recent *ex-vivo* data indicating that the microenvironment profoundly affects the quality of terminal megakaryocyte maturation (7, 35) and provide the first morphological evidence that the microenvironment may also affect the quality of megakaryocytes that mature *in vivo*.

The most striking difference observed between megakaryocytes from BM and spleen of MF patients is the maturation profile. By contrast with the immature morphology of megakaryocytes from BM, the maturation profile of cells from spleen is grossly normal with a nearly normal cellular distribution along the maturation classes and a good proportion of cells at Stage III presenting properly developed DMS and platelet territories. Furthermore, in spleen, Stage III megakaryocytes are frequently localized with platelets in proximity of blood vessels, providing circumstantial evidence for the hypothesis that these cells are capable to produce platelets.

It has been hypothesized that in MF megakaryocyte maturation is halted by a RSP14 signature induced by the driver mutations which reduces the number of ribosomes and their ability to bind and translate GATA1 mRNA, greatly reducing the content of this transcription factor that is indispensable for proper lineage maturation (19, 36). The fact that the maturation of megakaryocytes in the spleen from these patients is normal suggested to us that additional abnormalities cooperate with GATA1 deficiency in halting the maturation of megakaryocytes in MF BM. This hypothesis is tested by the more comprehensive comparison of TEM abnormalities present in megakaryocytes from the two organs discussed below.

Terminal megakaryocyte maturation greatly increases the cellular demand for lipids that are necessary to synthesize new membranes in the amounts required for proper organization of the DMS. Therefore, in addition to transcription factors, this process is limited by lipid bioavailability. Consistent with our previous reported that impaired cholesterol homeostasis is the top eight hit of the expression signature of MF BM (33), our TEM observations indicate that the membranes of megakaryocytes from MF BM present abnormalities (thick plasma membrane and nuclear membrane with large pores) usually associated with poor lipid content. In addition, these cells present in their cytoplasm numerous glycosomes, suggesting that their glycogen metabolism is skewed toward accumulation of acid-insoluble polyglucosan. The metabolisms of lipids and glycogen, the two major storage forms of cell energy, are tightly interrelated as demonstrated by the observation that accumulation of glycogen in the liver elicits a positive feedback loop involving mTORC1 that increases the expression of lipogenic genes leading to coordinated synthesis of glycogen and lipids (37). The fact that megakaryocytes from MF BM contain high numbers of large, electron-dense glycosomes suggests that these cells store great levels of glycogen in its insoluble polyglucosan form that is not readily available for energy homeostasis. Further support for this hypothesis comes from a re-analyses of the expression signature of MF BM (33) that identified modest fold changes above threshold in the expression of several genes of the glycogen pathway, four of which (*PHKA2*, *PHKB*, and *PYGL* down-regulated whereas *PRDM8* upregulated) mutated in inherited disorders associated with increased glycogen storage (38–44) (**Table S2**, **Figure S3**). Upregulation of *PRDM8* expression is particularly intriguing because gain of function mutations of this gene are associated with the neurodegenerative Lafora disease (30, 41) which is characterized by the presence in the neurons of specific polyglucosan-rich glycosomes, defined Lafora body, similar in morphology to some of the glycosomes identified by us in the malignant megakaryocytes from MF BM. In conclusion, the expression signature and the TEM observations support the hypothesis that the glycogen pathway in megakaryocytes from MF BM is skewed toward the formation of acid-insoluble glycosomes, making the energy of the cells exquisitely depend on lipid consumption and reducing the lipid pool devoted to membrane biosynthesis.

A recent single cell expression profiling has indicated that megakaryocytes present in BM exert distinct biological functions as they mature (45): the most immature megakaryocytes expression high levels of factors which regulate the hematopoietic stem cells, such as TGF-β and IGF1, suggesting that these cells exert the function of negative regulators of the hematopoietic stem cell niche. More mature megakaryocytes express genes predicting the function of activators of the inflammatory response (high levels of *CTSS* and *ITGAM*) and finally only the most mature cells express genes (*GPIbA, ITGB3*) necessary to produce platelets (45). According to this functional classification, the malignant megakaryocytes in the BM of MF patients, which are mostly at Stage I and II of maturation, are enriched for negative regulators of the hematopoietic stem cell niche and for promoters of inflammation, providing further support for the hypothesis that these malignant cells drive the hematopoietic failure in the BM (26).

Abnormalities unique to the spleen are represented by presence of low number of morphologically “altered” mitochondria. The fact that these abnormalities are not present in megakaryocytes from BM is consistent with the observation that the signature of the BM from MF patients does not contain great alterations in genes encoding for proteins involved in mitochondrial function (**Table S3**). Unfortunately, since the expression signature of the MF spleen has not been determined as yet, we have limited clues on the mechanisms that induce these abnormalities in the malignant megakaryocytes from this organ. Since the MF spleens contain great levels of reactive oxygen species (ROS) (46), possibly produced by mitochondria which are also susceptible to oxidative damage (47), we suggest that increased oxidative stress is responsible for the mitochondria abnormalities observed in the malignant cells from this organ.

The hypothesis that lipid and energy metabolisms are impaired in MF is consistent with a recent report indicating that the *JAK2* mutation alters lipid and glucose metabolism in animal models of myeloproliferative neoplasms. Furthermore, the phenotype of this model is rescued both by high fatty acid diet supplementation and pharmacological inhibition of the glycolytic enzyme PFKFB3, overexpressed by malignant cells, in combination with ruxolitinib (48). Our results refine the conclusions of this study indicating that in MF patients’ lipid and energy metabolism are likely skewed, respectively, in megakaryocytes from BM and spleen. This knowledge, in turn, suggests that high fat diet ameliorates disease manifestation by rescuing the membrane biosynthetic defect and consequently halting the maturation block of megakaryocytes from BM, thus depleting this organ from the cells that drive the manifestation of the disease.

The descriptive nature of TEM observations poses intrinsic limitations to the mechanistic insights provided by our study. Historically, however, TEM observations, by depicting the reality of organ malformations, have been instrumental to drive investigations on the pathobiology of numerous diseases, including MF (11, 20, 22). Our results continue in this tradition by providing information that highlights the importance of performing additional studies to clarify the role played by metabolic alterations of malignant megakaryocytes, possibly induced by microenvironment cues, in the pathobiology of MF. Ethical and technical limitations make it impossible to perform these studies using primary megakaryocytes prospectically isolated from BM and spleen of MF patients. In fact, the underlying fibrosis challenges the quality of BM biopsies from these patients that are usually small that contain number of cells insufficient for any analyses in 50% of the cases (33). The ethical limitation is that the symptoms of only a minority of MF patients indicate the need for splenectomy. As a consequence, although the study was conducted in collaboration with the two largest MF centers in Italy, it took us more than 3 years to collect specimens from the number of patients included in this study. It is predicted that it will be impossible to obtain additional MF spleen samples in the future because these symptoms are currently treatable with ruxolitinib (49). Usually, these challenges are addressed by analyzing as surrogate models megakaryocytes expanded *ex vivo* from CD34+ cells present in great numbers in these patients’ blood. However, the poor fidelity existing between megakaryocyte maturation *in vitro* and *in vivo* (7, 35) suggests that results obtained with megakaryocytes expanded *ex-vivo* may not completely reproduce the *in vivo* situation. Luckily megakaryocytes from the BM and spleen of animal models, such as the *Gata1^low^* mouse, express TEM abnormalities superimposable to those described here for MF patients [(36) and results not shown]. It is predicted that metabolic studies in these models will be instrumental to clarify the effects of the organ microenvironment on megakaryocyte maturation in MF.

In conclusion, megakaryocytes from BM and spleen of MF patients present unique morphological alterations suggesting that the two organs experience different metabolic abnormalities: those from BM show glycosomes and membrane alterations suggestive of inefficient carbohydrate and lipid metabolism while those from spleen express mitochondria abnormalities suggesting impaired aerobic bioenergetics. Both metabolic abnormalities represent possible novel therapeutic targets for MF.

## Data Availability Statement

The datasets presented in this study can be found in online repositories. The names of the repository/repositories and accession number(s) can be found below: https://www.ncbi.nlm.nih.gov/geo/, http://www.ncbi.nlm.nih.gov/geo/query/acc.cgi?acc=GSE124281.

## Ethics Statement

The studies involving human participants were reviewed and approved by the institutional review board of Policlinico San Matteo, Pavia, Italy (Authorization No. 20110004143, Sept 26, 2011) and are compliant with the Declaration of Helsinki for Studies Involving Human Subjects. The patients/participants provided their written informed consent to participate in this study.

## Author Contributions

MZ performed the ultrastructural analyses and wrote the manuscript. VR, AV, PG, and GB provided BM and spleen from the patients, and HC and wrote the manuscript. MG and MM performed the statistical analyses of the ultrastructural data and/or the pathway analyses of microarrays and wrote the manuscript. AM designed the study, interpreted data, and wrote the manuscript. All authors contributed to the article and approved the submitted version.

## Funding

This study was supported by grants from the National Cancer (P01-CA108671 ARM), National Heart, Lung and Blood Institute (1R01-HL116329, ARM), Associazione Italiana Ricerca Cancro (AIRC IG17608 and IG23525, ARM, and AIRC5×1000 project #21267, Metastatic disease: the key unmet need in oncology” to MYNERVA, MYeloidNEoplasms Research Venture AIRC) and by Accelerator Award Project funded through a partnership between Cancer Research UK, Fondazione AIRC and FundacionCientifica de la Asociacion Espanola Contra el Cancer to AMV.

## Conflict of Interest

The authors declare that the research was conducted in the absence of any commercial or financial relationships that could be construed as a potential conflict of interest.
