# Supplementary material for: Shared and Distinctive Ultrastructural Abnormalities Expressed by Megakaryocytes in Bone Marrow and Spleen From Patients With Myelofibrosis
Source: Front Oncol. 2020 Nov 16;10:584541. doi: 10.3389/fonc.2020.584541 (PMC7701330; doi:10.3389/fonc.2020.584541)
Supplement: Supplementary file 1 [file DataSheet_1.pdf]

Supplementary Material

Tables S1: List of the genes discussed in the paper. Abbreviation, name and identification number (Enzyme Commission (EC)number or UniProt ID) are reported. mRNA are identified by their Online Mendelian Inheritance in Man (OMIM) number (from https://www.uniprot.org/).

Gene	Protein	ID
AGL/GDE	Glycogen debranching enzyme	EC:2.4.1.25<br>EC:3.2.1.33
AKT1	AKT Serin/Threonin Kinase 1<br>(RAC-alpha serine/threonine-protein kinase or PKB)	EC:2.7.11.1
AKTIP	AKT interacting protein	Q9H8T0
ALDO	Fructose-bisphosphate aldolase	EC:4.1.2.13
ALDO A	Aldolase, muscle form	EC:4.1.2.13
ALDO C	Aldolase, brain form	EC:4.1.2.13
ATP5F1B	ATP synthase subunit beta	EC:7.1.2.2
CARL	Calreticulin	P27797
CIAO1	Cytosolic Iron-Sulfur Assembly Component 1	O76071
CLUH	Clustered mitochondria protein	O75153
CTSS	Cathepsin S	EC 3.4.22.27<br>EC 3.4.22
EPM2A	Epilepsy, Progressive Myoclonus Type 2A<br>(Laforin)	EC:3.1.3.16<br>EC:3.1.3.48
EPM2AIP1	EPM2A Interacting Protein 1	Q7L775

EPM2B	Epilepsy, Progressive Myoclonus Type 2A	Malin
FBP	Fructose-Bisphosphatase 1	EC 3.1.3.11
G6PC	Glucose-6-Phosphatase Catalytic Subunit (Glucose-6-phosphatase)	EC:3.1.3.9
GAA	Lysosomal Alpha Glucosidase	EC:3.2.1.20
GATA1	GATA Binding Protein 1	P15976
GBE1	1,4-Alpha-Glucan Branching Enzyme 1	EC:2.4.1.18
GLUT2	Solute Carrier Family 2 (Facilitated Glucose Transporter), Member 2	P11168
GP1BA	Glycoprotein Ib Platelet Subunit Alpha	P07359
GPI	Glucose-6-Phosphate Isomerase	EC:5.3.1.9
GSK3B	Glycogen Synthase Kinase 3 Beta	EC:2.7.11.26
GYG1	Glycogenin1	EC 2.4.1.186
GYS	Glycogen Synthase	EC 2.4.1.11
HK2	Hexokinase2	EC:2.7.1.1
HK3	Hexokinase3	EC:2.7.1.1
IGF1	Insulin Like Growth Factor1	P05019
ITGAM	Integrin Subunit Alpha M	P11215
ITGB3	Integrin Subunit Beta 3	P05106
JAK 2	Janus Kinase 2	O60674

MPL	MPL Proto-Oncogene, Thrombopoietin Receptor	P40238
MTFR1	Mitochondrial fission regulator 1	Q15390
mTORC1	Mechanistic Target of Rapamycin Complex 1	EC 2.7.11.1
OXA1L	Mitochondrial inner membrane protein	Q15070
PARP1	Poly [ADP-ribose] polymerase 1	EC 2.4.2.-
PFK	Phosphofructokinase	EC:2.7.1
PFKFB3	6-Phosphofructo-2-Kinase/Fructose-2,6-Biphosphatase 3	EC: 3.1.3.46
PGAM1	Phosphoglycerate Mutase 1	EC:5.4.2.11<br>EC:5.4.2.4
PGM1	Phosphoglucomutase-1	EC 5.4.2.2
PHKA2	Phosphorylase Kinase Regulatory Subunit Alpha 2	EC:2.7.11.19
PHKB	Phosphorylase Kinase Regulatory Subunit Beta	EC:2.7.11.19
PINK1	Serine/threonine-protein kinase PINK1	EC 2.7.11.1
PRDM8	PR/SET Domain 8	EC 2.1.1.-
PYGB	Glycogen Phosphorylase, Brain Form	EC 2.4.1.1
PYGL	Glycogen phosphorylase, liver form	EC:2.4.1.1
RHOT2	Mitochondrial Rho GTPase 2	EC 3.6.5.-
SGA1	Glucoamylase	EC:3.2.1.3
SGK3	Serine/threonine kinase 3	EC:2.7.11.1
SLC25A37	Solute Carrier Family 25 Member 37	Q9NYZ2
SLC25A39	Solute Carrier Family 25 Member 39	Q9BZJ4

TGFB	Transforming growth factor-β	P01137
UGP2	UDP-Glucose Pyrophosphorylase 2	EC:2.7.7.9
UQCRH	Cytochrome b-c1 complex subunit 6	P07919

Table S2: Expression signature of the glycogen pathway in BM from myelofibrosis patients (the expression signature is from (1))

Level of expression: HIGH → LOW

GSD	Gene	Protein	ID	Fold Change	P value (FDR)
	PFKFB3	6-Phosphofructo-2-Kinase/Fructose-2,6-bisphosphatase-3	3.1.3.46	1.998	0.195
*	**PRDM8**	**PR domain containing protein-8**	**2.1.1.-**	**1.544**	**0.226**
	GPI	Glucose-6-Phosphate Isomerase	5.3.1.9	1.206	0.119
	G6PC	Glucose-6-Phosphatase Catalytic Subunit	3.1.3.9	1.197	0.082
	PFKM	6-Phosphofructokinase, muscle type	2.7.1.11	1.183	0.069
	PFKP	6-Phosphofructokinase, platelet type	2.7.1.11	1.177	0.290
	GSK3B	Glycogen Synthase Kinase-3β	2.7.11.26	1.147	0.369
	PYGB	Glycogen Phosphorylase, brain form	2.4.1.1	1.129	0.184
	HK2	Hexokinase-2	2.7.1.1	1.112	0.889
	AKT1	AKT Serin/Threonin Kinase-1	2.7.11.1	1.088	0.553
	ALDOC	Aldolase, brain form	4.1.2.13	1.036	0.833
	UGP2	UDP-Glucose Pyrophosphorylase-2	2.7.7.9	0.969	0.900
	ALDOA	Aldolase, muscle form	4.1.2.13	0.936	0.886
	GBE1	1,4-α-Glucan Branching Enzyme-1	2.4.1.18	0.916	0.232
	GAA	Glucosidase-α, Acid	3.2.1.20	0.883	0.426
	EPM2AIP1	EPM2A Interacting Protein 1	Q7L775	0.869	0.446
*	PHKB	Phosphorylase Kinase Regulatory Subunit-β	2.7.11.19	0.818	0.008
	GYG1	Glycogenin-1	2.4.1.186	0.816	0.088
	SGK3	Serum- and glucocorticoid-inducible protein kinase-3	2.7.11.1	0.766	0.008
	AKTIP	AKT interacting protein	Q9H8T0	0.756	0.212
	PGM1	Phosphoglucomutase-1	5.4.2.2	0.753	0.008
*	PYGL	Glycogen Phosphorylase, liver form	2.4.1.1	0.748	0.118
*	PHKA2	Phosphorylase Kinase Regulatory Subunit-α2	2.7.11.19	0.744	0.067
	HK3	Hexokinase-3	2.7.1.1	0.645	0.184
	PGAM1	Phosphoglycerate Mutase-1	5.4.2.11	0.631	0.133

* associated with inherited glycogen storage diseases

*Indicates genes mutated in inherited diseases associated with abnormal glycogen storage. Loss of function of *PHKB* and *PHKA2* are observed in Glycogen Storage Disease Type IX(2-4). Loss of function of *PYGL* is associated with glycogenosis type VI and its deletion induces a Glycogen Storage Disease Type VI phenotype with liver fibrosis in mice(5-6). PRDM8 interacts with the laforin/malin phosphatase complex and causes sequestration of the two proteins to the nucleus(7). In Lafora disease, the gain-of-function *PRDM8*F261L mutation encodes a protein that sequesters laforin and malin in the nucleus, leading to their cytoplasm deficiency and increasing the phosphorylation of glycogen into insoluble polyglucosan(8) (see also **Figure S3**).

Table S3: Expression signature of the mitochondrial homeostasis in BM from myelofibrosis patients. The expression signature is from (1)**.**

Level of expression: HIGH – LOW

Gene	Protein	ID	Fold Change	P value (FDR)
SLC25A37	Solute Carrier Family 25 Member 37	Q9NYZ2	2.385	0.363
SLC25A39	Solute Carrier Family 25 Member 39	Q9BZJ4	2.331	0.263
PINK1	Serine/threonine-protein kinase PINK1*	2.7.11.1	1.511	0.214
CLUH	Clustered mitochondria protein	O75153	1.306	0.078
RHOT2	Mitochondrial Rho GTPase 2*	3.6.5.-	1.224	0.426
ATP5F1B	ATP synthase subunit beta*	7.1.2.2	1.216	0.088
UQCRH	Cytochrome b-c1 complex subunit 6	P07919	1.115	0.759
MTFR1	Mitochondrial fission regulator 1	Q15390	1.084	0.427
PARP1	Poly [ADP-ribose] polymerase 1*	2.4.2.-	1.043	0.863
OXA1L	Mitochondrial inner membrane protein	Q15070	0.696	0.008
CIAO1	Cytosolic Iron-Sulfur Assembly Component 1	O76071	0.647	0.008

* ID codes for enzyme proteins according to the E.C. numerical classification scheme

Supplementary Figures

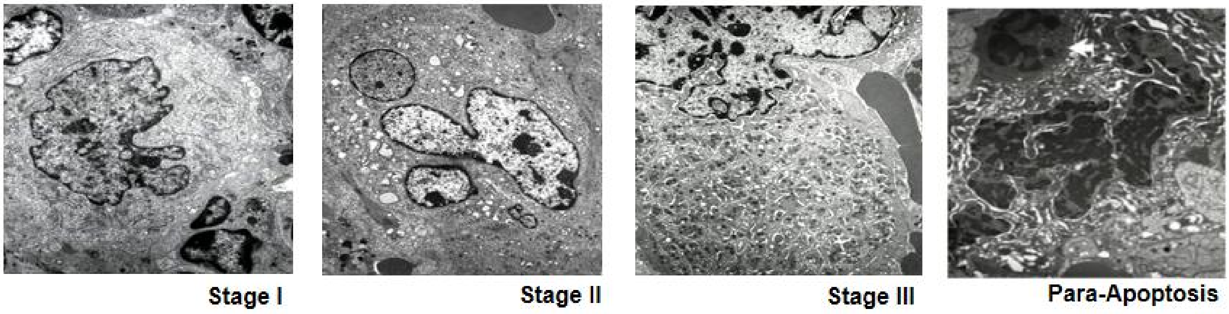

Figure S1: Morphological criteria used to define the maturation (stage I, II and III) of normal megakaryocytes and to identify megakaryocytes in para-apoptosis by transmission electron microscopy (see(9) for further detail). Megakaryocytes at Stage 0 are not shown because these cells may not be univocally identified by this technique. Magnification 3000x**.**

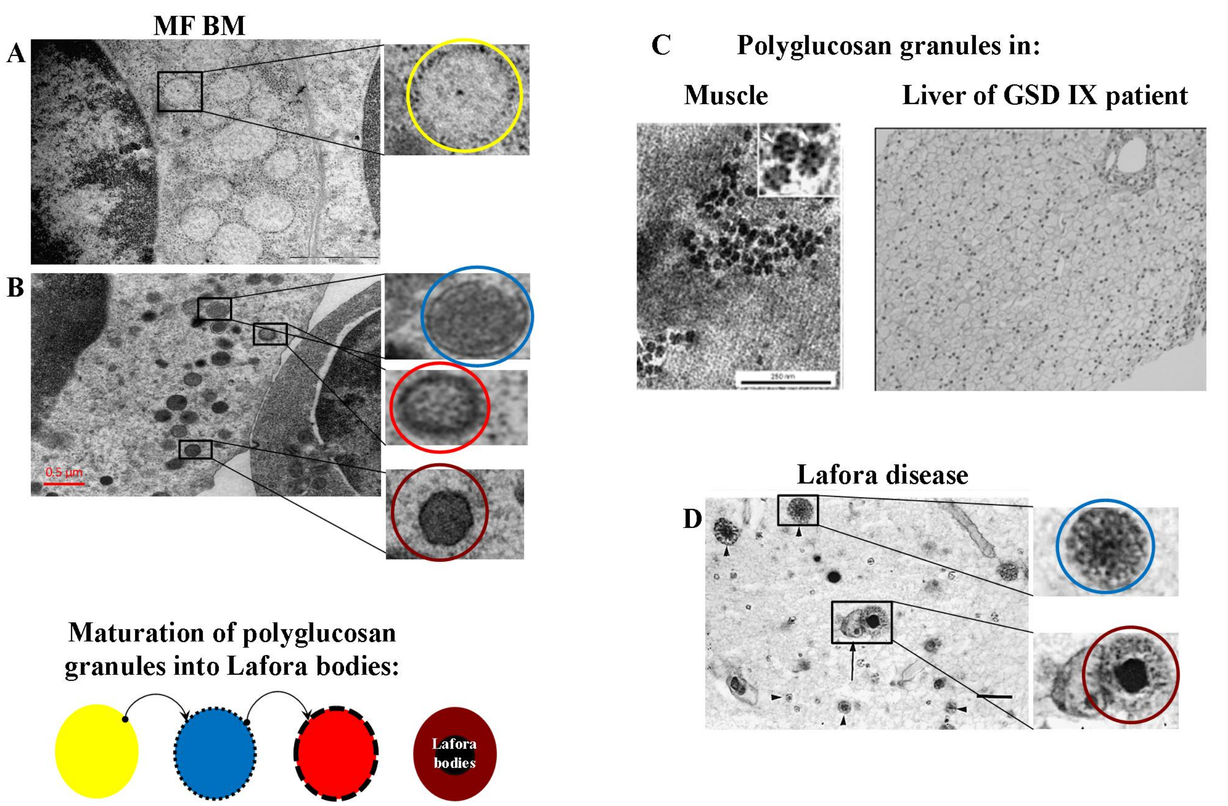

Figure S2: Comparison of the glycosomes found in the cytoplasm of two representative megakaryocytes from bone marrow (BM) of myelofibrosis (MF) patients (A,B) with those described in the muscle of normal donors (panel on the left in C) or in the liver of patients with glycogen storage disease IX (panel on the right in C) or in the brain of Lafora disease (D). A diagram showing the maturation of polyglucosan granules in glycosomes based on the progressive increase of their number and electron density is reported on the bottom. Low electron dense acid-labile granules containing glycogen and regulatory proteins may (A) or may not (not shown) organize themselves as rims delimiting discrete cytoplasmic areas, the immature glycosome A). These granules are turned by phosphorylation into heavy-electron dense acid-insoluble polyglucosan granules (right panel in C) which, on the basis of their density, may give rise to intermediate (blue circles) and then mature (red circles) glycosomes. Acid-insoluble polyglucosan molecules associated with glycogen storage diseases may accumulate as isolated granules in the cytoplasm (right panel in C) or coalesce into packed glycosomes such as those found in the Lafora disease (D). The cytoplasm of megakaryocytes from BM of myelofibrosis patients contain glycosomes at all stages of maturation including few cases (circled in purple) resembling Lafora bodies. Scale bar 0.5 µm, as indicated. C and D are published by permission from(10-12).

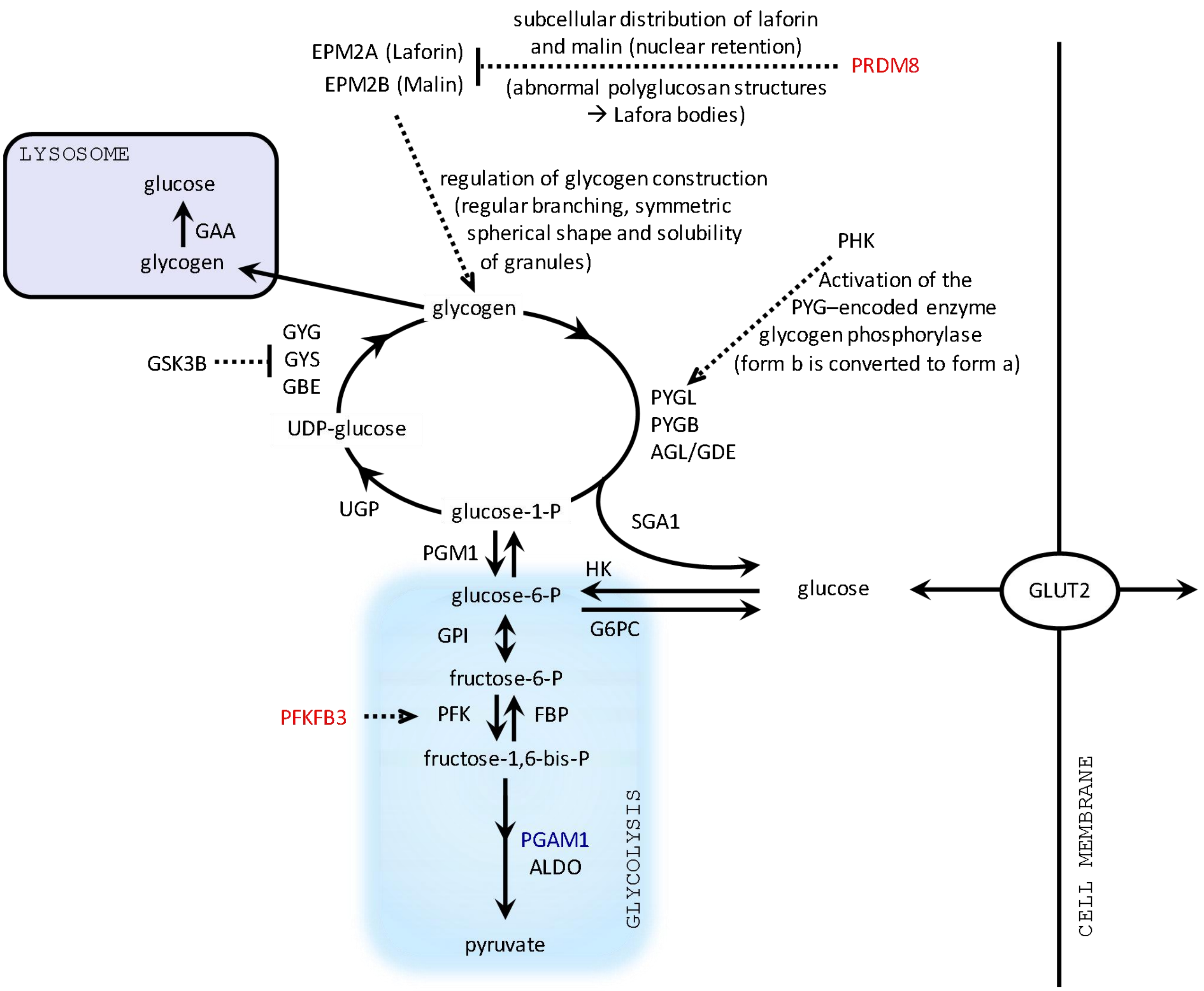

Figure S3: **Diagram of the biochemical pathways involved in glycogen synthesis/degradation and polyglucosan accumulation.** Genes expressed at altered levels in the expression signature of BM from myelofibrosis patients are indicated in red (over-expressed) and blue (under-expressed) fonts, respectively. Biosynthesis of acid-soluble glycogen granules results from dimerization and autoglycosylation catalyzed by glycogenin (GYG) and by the coordinated activity of glycogen synthase (GYS) and of the branching enzyme (GBE) which provides to the complex a 3D-spherical and branched structure and the hydrophilic surface necessary for solubility in the cytoplasm. The protein laforin and malin regulate the solubility of the granules by assuring that the branching of the polyglucosan molecule is regular. PRDM8, the early-onset Lafora disease protein(10) overexpressed also in myelofibrosis BM (**Table S2**), favors irregular branching by inducing nuclear retention of laforin and malin(11-13).

References for the Supplementary information

1. Ishikawa G, Fujiwara N, Hirschfield H, Varricchio L, Hoshida Y, Barosi G, Rosti V, Padilla M, Mazzarini M, Friedman SL, et al. Shared and Tissue-Specific Expression Signatures between Bone Marrow from Primary Myelofibrosis and Essential Thrombocythemia. *Exp Hematol* (2019) **79**:16-25.e3. doi:10.1016/j.exphem.2019.10.001

2. Beauchamp NJ, Dalton A, Ramaswami U, Niinikoski H, Mention K, Kenny P, Kolho K-L, Raiman J, Walter J, Treacy E, et al. Glycogen storage disease type IX: High variability in clinical phenotype. *Mol Genet Metab* (2007) **92**:88–99. doi:10.1016/j.ymgme.2007.06.007

3. Choi R, Park HD, Kang B, Choi SY, Ki CS, Lee SY, Kim JW, Song J, Choe YH. PHKA2 mutation spectrum in Korean patients with glycogen storage disease type IX: Prevalence of deletion mutations. *BMC Med Genet* (2016) **17**: 33 doi:10.1186/s12881-016-0295-1

4. Fu J, Wang T, Xiao X. A novel PHKA2 mutation in a Chinese child with glycogen storage disease type IXa: a case report and literature review. *BMC Med Genet* (2019) **20**:56. doi:10.1186/s12881-019-0789-8

5. Burwinkel B, Bakker HD, Herschkovitz E, Moses SW, Shin YS, Kilimann MW. Mutations in the Liver Glycogen Phosphorylase Gene (PYGL) Underlying Glycogenosis Type VI (Hers Disease). *Am J Hum Genet* (1998) **62**:785–791. doi:10.1086/301790

6. Wilson LH, Cho J, Estrella A, Smyth JA, Wu R, Chengsupanimit T, Brown LM, Weinstein DA, Lee YM. Liver Glycogen Phosphorylase Deficiency Leads to Profibrogenic Phenotype in a Murine Model of Glycogen Storage Disease Type VI. *Hepatol Commun* (2019) **3**:1544–1555. doi:10.1002/hep4.1426

7. Turnbull J, Tiberia E, Striano P, Genton P, Carpenter S, Ackerley CA, Minassian BA. Lafora disease. *Epileptic Disord* (2016) **18**:38–62. doi:10.1684/epd.2016.0842

8. Turnbull J, Girard JM, Lohi H, Chan EM, Wang P, Tiberia E, Omer S, Ahmed M, Bennett C, Chakrabarty A, et al. Early-onset Lafora body disease. *Brain* (2012) **135**(9): 2684-2698 doi:10.1093/brain/aws205

9. Centurione L, Di Baldassarre A, Zingariello M, Bosco D, Gatta V, Rana RA, Langella V, Di Virgilio A, Vannucchi AM, Migliaccio AR. Increased and pathologic emperipolesis of neutrophils within megakaryocytes associated with marrow fibrosis in GATA-1low mice. *Blood* (2004) **104**:3573–3580. doi:10.1182/blood-2004-01-0193

10. Cavanagh JB. Corpora-amylacea and the family of polyglucosan diseases. *Brain Res Brain Res Rev* (1999) **29**:265–95. doi:10.1016/s0165-0173(99)00003-x

11. Prats C, Graham TE, Shearer J. The dynamic life of the glycogen granule. *J Biol Chem* (2018) **293**:7089–7098. doi:10.1074/jbc.R117.802843

12. Kim JA, Kim JH, Lee BH, Kim G, Shin YS, Yoo H, Kim KM. Clinical, Biochemical, and Genetic Characterization of Glycogen Storage Type IX in a Child with Asymptomatic Hepatomegaly. *Pediatr Gastroenterol Hepatol Nutr.* (2015) **18**:138–143.

13. Montori-Grau M, Guitart M, Lerin C, Andreu AL, Newgard CB, García-Martínez C, Gómez-Foix AM. Expression and glycogenic effect of glycogen-targeting protein phosphatase 1 regulatory subunit GL in cultured human muscle. *Biochem J* (2007) **405**:107–113. doi:10.1042/BJ20061572
